# Epithelial Mesenchymal Transition in Embryonic Development, Tissue Repair and Cancer: A Comprehensive Overview

**DOI:** 10.3390/jcm7010001

**Published:** 2017-12-22

**Authors:** Do Hyung Kim, Tiaosi Xing, Zhibin Yang, Ronald Dudek, Qun Lu, Yan-Hua Chen

**Affiliations:** 1 Department of Anatomy and Cell Biology, East Carolina University, Greenville, NC 27834, USA; doh.kim04@gmail.com (D.H.K.); xingt12@students.ecu.edu (T.X.); yangz15@ecu.edu (Z.Y.); dudekr@ecu.edu (R.D.); luq@ecu.edu (Q.L.); 2 The Sidney Kimmel Comprehensive Cancer Center, Johns Hopkins University, Baltimore, MD 21287, USA; 3 Leo Jenkins Cancer Center, The Brody School of Medicine, East Carolina University, Greenville, NC 27834, USA

**Keywords:** epithelial mesenchymal transition, embryonic development, tissue repair, tumor formation and progression, stem cells

## Abstract

The epithelial mesenchymal transition (EMT) plays a central role in both normal physiological events (e.g., embryonic development) and abnormal pathological events (e.g., tumor formation and metastasis). The processes that occur in embryonic development are often reactivated under pathological conditions such as oncogenesis. Therefore, defining the regulatory networks (both gene and protein levels) involved in the EMT during embryonic development will be fundamental in understanding the regulatory networks involved in tumor development, as well as metastasis. There are many molecules, factors, mediators and signaling pathways that are involved in the EMT process. Although the EMT is a very old topic with numerous publications, recent new technologies and discoveries give this research area some new perspective and direction. It is now clear that these important processes are controlled by a network of transcriptional and translational regulators in addition to post-transcriptional and post-translational modifications that amplify the initial signals. In this review article, we will discuss some key concepts, historical findings, as well as some recent progresses in the EMT research field.

## 1. Introduction

Epithelial and mesenchymal cell types were first recognized in the late Nineteenth Century based on cell shape and organization during embryonic development. Although the inter-conversion between epithelial cells and mesenchymal cells was initially described by Frank Lillie in 1908 [1], the concept of an epithelial cell to mesenchymal cell transition (EMT) as a distinct cell process was not put forward until the 1980s by Greenburg and Hay [2]. Since then, the importance of EMT as a distinct cell process has been well recognized as the EMT has been implicated in a number of different biological processes, which include: embryonic development, embryonic stem cell differentiation, induction of pluripotency, tissue repair, wound healing, stem cell behavior and in pathological conditions such as tissue/organ fibrosis, cancer stem cell behavior and cancer progression [3,4].

The inter-conversion between epithelial cells and mesenchymal cells is a highly conserved and reversible cell process whereby polarized, immotile epithelial cells extend filopodia from their basal surface and give rise to migrating mesenchymal cells. After undergoing an EMT, cells acquire migratory and invasive properties that allow their migration through the extracellular matrix (ECM). The activation of the EMT process depends on microenvironmental signals and on EMT-activating signals that interact with epigenetic regulators. These epigenetic regulators control the expression of proteins involved in several areas, which include: cell polarity, cell-cell adhesion, cell-cell contact, cytoskeleton degradation and ECM degradation. In addition, these epigenetic regulators cause the repression of key epithelial genes and the acquisition of migratory and invasive properties [5,6,7].

Recent studies have indicated that the EMT process plays a critical role in tumor progression, malignant transformation and resistance to therapy by acting through a variety of mechanisms, which produce the following effects: endowing the cell with migratory and invasive properties, inducing stem cell properties, preventing apoptosis and senescence and contributing to immunosuppression [4].

This review begins with: (i) a discussion of key concepts involving epithelium and mesenchyme along with an EMT classification; (ii) next, we discuss EMT and embryonic development specifically focusing on the role of EMT in gastrulation and neural crest formation, along with the reverse process called mesenchymal to epithelial transition (MET); (iii) we then explore EMT in wound healing, tissue regeneration and organ fibrosis; (iv) we also discuss the clinically-relevant topic of EMT and cancer, specifically focusing on the role of EMT in cancer cell survival, disruption of cell junctions and polarity, circulating tumor cells and drug resistance; (v) finally, we conclude with a discussion of EMT and gene expression, EMT and signaling pathways, EMT and microRNAs, EMT and stem cells and EMT and autophagy.

## 2. Key Concepts and EMT Classification

An EMT can be defined as a process that produces a complete loss of epithelial traits by the former epithelial cells accompanied by the acquisition of mesenchymal traits [8]. More specifically, epithelial cell layers lose their polarity and cell-cell contact while undergoing a dramatic cytoskeletal remodeling [4]. A hallmark of the EMT process is the acquisition of the ability to migrate and invade the ECM as single cells [3]. Although an EMT follows a common and conserved program, it also has an inherent flexibility and some variation, which depends on the cell type, tissue context and EMT-activating signals [9]. An EMT is reversible, and as such, the reverse process is termed as MET, which also occurs both during embryonic development and disease pathogenesis. This reversibility underscores the plasticity of certain embryonic cells and the plasticity of adult cells that are involved in disease pathogenesis [3].

Due to the flexibility of an EMT, many other terms besides “transition” have been used in the past to describe this process, for example, an epithelial-mesenchymal “transformation”, “interaction” or “trans-differentiation”. The term “transformation” describes a permanent alteration of the cell. The term “interaction” refers to proximate cell signaling between the epithelium and adjacent mesenchyme as seen in ameloblasts and dental papillae mesenchymal cells changing into odontoblasts. The term “trans-differentiation” refers to differentiated cells that change into other differentiated cells. The term “transition” is now the preferred and more appropriate term to describe an EMT since this process is reversible and mesenchymal cells can revert or “transition back” to epithelial cells via an MET [10,11].

Depending on the tissue context or EMT-activating signals, epithelial cells may lose only some of their traits or may show a combination of epithelial and mesenchymal cell traits both of which are considered a “partial” EMT. An EMT is a dynamic process with the existence of intermediate states as demonstrated by the identification of a transitioned or “EMTed” phenotype (either partial or complete) in circulating tumor cells [7,12,13,14]. The cells that are arrested in or transitioning through these intermediate (or metastable) states of an EMT are often call “fused cells” since they have attributes of both an epithelial and mesenchymal phenotype and also exhibit stem cell-like properties [7,15]. These “fused cells” also display a high degree of plasticity between the epithelial and mesenchymal phenotypes, which plays a critical role in cancer metastasis [7,16,17,18].

Although epithelial cells and mesenchymal cells show distinct morphological and functional differences, the exact criteria (i.e., an all-encompassing checklist) to accurately distinguish epithelial cells from mesenchymal cells remains controversial. In general, the EMT-induced changes in cell phenotype include the following: (1) a morphological change from epithelial cells arranged in a cobblestone-like monolayer with apical-basal polarity to spindle-shaped mesenchymal cells with a migratory pseudopodium arranged in a dispersed manner; (2) a change in differentiation markers from cell-cell junctional proteins and cytokeratin intermediate filaments in epithelial cells to fibronectin and vimentin intermediate filaments in mesenchymal cells; (3) a functional change associated with the conversion of stationary, epithelial cells to motile, mesenchymal cells that can migrate through the ECM [8,19]. However, there are EMT-induced changes that are not invariably observed, which include: (1) downregulation of epithelial gene expression and upregulation of mesenchymal gene expression; (2) increased number of cell protrusions and motility; (3) resistance to senescence and apoptosis [3].

Epithelial cells are highly polarized with respect to their apical border. Epithelial cells form single or multiple layers that are tightly connected on their lateral border by specialized junctions, including tight junctions, adherens junctions, desmosomes and gap junctions. The adherens junction plays a critical role in maintaining an epithelial layer and involves the protein E-cadherin. E-cadherin induces the formation of both the adherens junction and the desmosome [20]. The loss of E-cadherin results in the disappearance of catenins from junctions and all epithelial traits. During an EMT, epithelial cells lose their cell-cell junctions, lose their apical-basal polarity, undergo cytoskeletal remodeling, alter signaling pathways that define cell shape and reprogram gene expression. All of the above-mentioned changes increase cell motility and contribute to the development of a phenotype that can migrate through the ECM [1].

In contrast to epithelial cells, mesenchymal cells rarely establish the direct contact with nearby mesenchymal cells and migrate through the ECM whether the matrix is constructed by epithelial cells or mesenchymal cells themselves [8]. Mesenchymal cells cannot be identified based on the presence of stress fibers since cells with stress fibers are abnormal cells that do not migrate through the ECM [21,22]. Stress fibers are more clearly observed in cultures. The identity of a mesenchymal cell should be based on its morphology, invasive motility and secretory capability. A typical mesenchymal cell exhibits a front-back polarity for cell locomotion, a trailing pseudopodium and an active front end that contains a Golgi complex. During an EMT, cells reorganize the cortical actin cytoskeleton, which enables the cells to change their shapes. Actin-rich membrane projections at the leading edge of the front end facilitate cell movement. These actin-rich membrane projections include sheet-like protrusions called lamellipodia and spike-like extensions called filopodia. Actin-rich invadopodia exert a proteolytic function that degrades the ECM, thus facilitating cell invasion [23]. These dynamic changes in the actin cytoskeleton are mediated by various regulatory proteins (e.g., Rho family small GTPases and moesin) and signaling mechanisms [24,25]. However, the exact molecular mechanisms involved in F-actin dynamics during an EMT remain as a focus of intensive research [26].

An EMT may occur in three distinct biological settings that result in vastly different outcomes. While the specific signals that delineate an EMT are not yet clear, it is now well accepted that functional distinctions are apparent based on the biological setting. Table 1 divides EMT into three distinct subtypes based on the biological setting in which the EMT occurs [27]. 

## 3. EMT during Embryonic Development

Gastrulation is an evolutionarily old process that establishes the three definitive germ layers of the embryo called ectoderm, mesoderm and endoderm forming the trilaminar embryonic disk. In contrast, neural crest cell formation is an evolutionary new process that forms, but not limited to, the skeletal and connective tissue components of the vertebrate head and the ganglia of the peripheral nervous system.

During embryonic development, sequential rounds of EMT and MET are needed for the final differentiation of specialized cell types and the formation of the three-dimensional structure of the organs. These sequential rounds are referred to as a primary EMT, secondary EMT and tertiary EMT [4].

A primary EMT occurs early in embryonic development, even before implantation (e.g., formation of the parietal endoderm in mice). The earliest primary EMT that occurs after implantation is the formation of mesoderm from the primitive ectoderm via gastrulation. The formation of mesoderm and neural crest represents key EMT processes, whereby the resulting mesenchymal cells and neural crest cells maintain multi-potentiality, which enables these cells to differentiate further into various cell types. A primary EMT is directed by epithelial morphogenesis whereby the epithelial cell layer undergoes de-epithelialization and then the epithelial cells undergo ingression. De-epithelialization occurs first followed by ingression and the adoption of basic mesenchymal characteristics like cell motility and migration.

A secondary EMT is a differentiation event that generates mesenchymal cells with a more restricted potential to differentiate into various cell types. In this regard, early mesodermal cells subdivide after gastrulation into axial, paraxial, intermediate and lateral mesoderm. These mesodermal subdivisions can then condense into transient epithelial tissues through an MET process, thereby forming the notochord, somites, precursors of the urogenital system and the somatopleure/visceropleure, respectively. The migratory neural crest cells travel stereotypical routes to their final destination and then differentiate into various cells types (e.g., neurons, bone and connective tissue). Some endodermal cells within their embryonic primordia exhibit morphological changes reminiscent of a secondary EMT, for example, the dissociation of endocrine cells within the pancreatic bud and the dissociation of hepatoblasts within the liver diverticulum [28].

A tertiary EMT is exemplified by embryonic heart formation that involves three successive cycles of an EMT and MET. Firstly, when cardiac mesodermal cells are generated by an EMT at gastrulation, cardiac progenitor cells in the visceropleure quickly become organized into a two-layered epithelium via an MET. Secondly, when the two cardiogenic areas fold ventral to the primitive foregut, mesenchymal cells delaminate from the epithelial layer to form a continuous sheet of cardiac mesoderm (sometimes called a heart-forming region) via an EMT. Thirdly, the mesenchymal cells within this continuous sheet of mesoderm give rise to the endothelial lining of the heart via an MET. In addition, the atrioventricular valvulo-septal complex (or atrioventricular septum) forms via an EMT as endothelial cells from the atrioventricular canal invade the cardiac jelly and form the dorsal and ventral endocardial cushions [29]. Finally, the fact that heart valve formation occurs in well-differentiated epithelium raises the possibility that an EMT may also be induced in well-differentiated adult tissues under certain physiological or pathological conditions, including tumor invasion and metastasis. 

The production of normal stem cells occurs through an EMT process in normal tissues. This phenomenon has been observed at the periphery of human embryonic stem (ES) cell clusters grown on Matrigel [30]. The pluripotent ES cells in the inner cell mass of the blastocyst demonstrate epithelial characteristics. During gastrulation, the pluripotent epithelial epiblast cells ingress to form the mesoderm through an EMT process [10]. Consequently, the EMT process that occurs during gastrulation represents an initial differentiation event that causes the generation of the three primary germ layers (i.e., ectoderm, mesoderm and endoderm) from pluripotent cells. ES cell or epiblast cell colonies grown in vitro give rise to cells located at the periphery of the cell colony that show a mesenchymal phenotype, which illustrates the importance of an EMT in early differentiation. The early, undifferentiated cells with a mesenchymal phenotype are characterized by a shift from E-cadherin expression to N-cadherin expression along with the expression of Snails, vimentin and metalloproteases. In addition, the early, undifferentiated cells with a mesenchymal phenotype retain the expression of several totipotent transcription factors (e.g., Oct4 and Nanog), which indicates that these cells can adopt a mesenchymal phenotype without losing their pluripotency [30]. 

### 3.1. EMT and Signaling Pathways in Gastrulation

The term gastrulation describes the formation of the three embryonic germ layers (i.e., ectoderm, mesoderm and endoderm) from the epiblast, which is the initial epithelial embryonic layer. The process of gastrulation essentially situates the ectoderm in the outer layer, the mesoderm in the middle layer and the endoderm in the inner layer of the embryo. The mesoderm and endoderm end up in their respective locations through a process called ingression. Ingression is an EMT process whereby individual cells from the outer epiblast detach and are internalized through the primitive streak.

In invertebrate gastrulation, Snail and Twist play a key role since they are required for the formation of an apical constriction, which then leads to ventral furrow formation and cell ingression [31]. Snail and Twist are transcription factors that repress E-cadherin and induce an EMT [32].

In vertebrate gastrulation, the canonical Wnt signaling pathway activated in the posterior region of the embryo plays a key role since this pathway is required for cells to ingress at the primitive streak. In addition, the Wnt signaling pathway renders the ingressing cells to respond to other extracellular signals that initiate an EMT [33]. 

In various species, Nodal and Vg1 play a key role in starting the gastrulation process. Nodal and Vg1 are members of the TGF-β superfamily that acts downstream of Wnt signaling [34]. In Wnt3-deficient mice, a primitive streak does not form so that the EMT associated with gastrulation does not occur [33,35]. In Wnt-overexpressing mice, multiple primitive streaks form [36].

Nodal and fibroblast growth factor (FGF) induce an EMT as cells ingress at the primitive streak during mouse gastrulation [37,38]. Nodal mutants result in the failure of complete gastrulation and can be rescued by transplantation of a few Nodal-expressing cells [39]. The cooperation of Nodal and Vg1 induces the formation of the primitive streak and cell ingression after Wnt signaling makes the epiblast competent to gastrulate [10]. The signaling through the FGF receptor (FGFR) maintains the EMT regulatory network [37,40]. In FGFR-deficient mice, the primitive streak and mesodermal cells form initially, but their activity is arrested [37].

### 3.2. EMT and Signaling Pathways during Neural Crest Formation

After gastrulation in vertebrates, neural crest cells migrate from the neural tube by following a classical EMT event where the cells within the neural tube changing from an epithelial phenotype into a migratory mesenchymal phenotype. These neural crest cells then delaminate from the neural folds and migrate into distinct regions of the embryo prior to their re-aggregation via an MET [10]. The MET gives rise to different derivatives, such as the ganglia of the peripheral nervous system and chromaffin cells of the adrenal medulla.

The signaling pathways employed during neural crest cell formation are similar to those pathways active at the primitive streak during gastrulation. The Wnt, FGF, retinoic acid and bone morphogenetic protein (BMP) signaling pathways are necessary for neural crest cell induction [41]. The canonical Wnt signaling pathway plays a role in the induction, stabilization and delamination of neural crest cell precursors; whereas, the non-canonical Wnt signaling pathway plays a role in contact inhibition of locomotion and directional movements of neural crest cells [42,43]. The role of TGF-β superfamily in neural crest formation involves BMPs. For example, BMP4 expression within the neural folds induces the epithelial cells into migratory neural crest cells through activating c-Myb that activates the transcription factors of msh homeobox 1 (Msx1) and Snail 2 [44,45]. BMPs may also modulate the timing of neural crest cell migration since Noggin (a BMP inhibitor) prevents this migration process [46]. Cv-2 (a vertebrate homologue of *Drosophila* crossveinless) regulates the BMP signaling pathway, thereby controlling the onset of neural crest cell migration in the trunk region, but not in the cranial region [47]. 

Sox E genes (*Sox8*, *Sox9* and *Sox10*), *Foxd3*, *Rhob* and *Snail2* genes play an important role in the neural crest formation, but may not be sufficient to induce a complete EMT [10,48,49]. Therefore, a combination action of several transcription factors is required to generate a complete neural crest cell EMT and the migration of neural crest cells from the neural tube. 

### 3.3. MET and Embryonic Development 

MET is the reversal of EMT process. Many studies over the years have shown that the ectopic expression of *E-cadherin* gene causes mesenchymal cells to transition into epithelial cells. The transfection of invasive corneal fibroblasts with the *E-cadherin* gene leads to their dramatic transition from a mesenchymal phenotype to an epithelial phenotype, specifically a stratified epithelium with desmosomes [50]. 

The best-studied MET event during embryonic development is the formation of the nephron epithelium in the kidney. During this MET process, nephric mesenchymal cells aggregate around individual branches of the ureteral bud, express laminin, polarize, develop cell-cell adhesions and finally differentiate into epithelial cells that form the renal tubules [51]. The ability of a mesenchymal cell to revert to an epithelial phenotype substantiates the existence of cell plasticity in the non-pathological condition and suggests that inter-conversion between mesenchymal and epithelial phenotypes can also occur in the pathological condition. 

It is worth pointing out that some adult (neoplastic) tissues, such as synovial sarcomas [52] and pleomorphic adenomas of the parotid gland [53], also display MET or EMT phenotype. Using matrix gene expression profiles as an additional important criterion, unequivocal epithelial and mesenchymal differentiation in pleomorphic adenomas was demonstrated [53].

## 4. EMT and Wound Healing, Tissue Regeneration and Organ Fibrosis

A Type II EMT occurs during wound healing, tissue regeneration and organ fibrosis. During wound healing and tissue regeneration, the EMT process begins as part of a repair-associated event that normally generates fibroblasts and other related cells in order to reconstruct tissues following injury [27]. Snail2 influences the “metastable state” in keratinocytes at the migratory front since Snail2 inactivation or overexpression compromises or accelerates wound healing, respectively [54]. In addition, ovarian surface epithelium undergoes an EMT process during the postovulatory wound healing in each menstrual cycle. Epidermal growth factor (EGF) induces this postovulatory wound healing through the activation of metalloproteases, ILK kinase and ERK kinases [55]. Finally, a subpopulation of tbx18-positive activated epicardial epithelium undergoes an FGF17b/FGFR2, FGFR4-dependent EMT process, which allows the epicardial epithelium to invade the regenerating myocardium and to facilitate myocardial neovascularization [56]. Consequently, the EMT process can be activated to repair tissue and re-establish tissue homeostasis.

Tissue fibrosis is basically an un-abated type of wound healing caused by persistent inflammation. A pathological EMT process resembles a non-pathological, physiological EMT process in that they are both governed by similar signaling pathways. Organ fibrosis occurs in a number of glandular epithelial tissues whereby inflammatory cells and fibroblasts release various inflammatory signals, as well as components of the ECM (e.g., collagen, laminin, elastin and tenascin). Cell tracing studies demonstrated that a significant portion of myofibroblasts arise from the conversion of epithelial cells via an EMT process [57]. In organ fibrosis, myofibroblasts produce an excessive amount of collagen, which compromises organ function and leads to organ failure. 

Fibroblast-specific protein 1 (FSP1), α-SMA and collagen I are reliable markers used to identify the mesenchymal cell phenotype generated by an EMT that occurs during organ fibrosis [58,59,60]. In addition to the above-mentioned markers, the discoidin domain receptor tyrosine kinase 2 (DDR2), vimentin and desmin are also reliable markers to identify epithelial cells that are undergoing an EMT in kidney, liver and intestines with inflammation [27]. Epithelial cells that are in the midst of undergoing an EMT associated with chronic inflammation not only exhibit epithelial-specific morphology and molecular markers (e.g., cytokeratin, E-cadherin), but also show concomitant FSP1 and α-SMA expression. These cells probably represent intermediate stages in the EMT process as both epithelial and mesenchymal markers are expressed concomitantly [27]. The cells within intermediate stages of an EMT eventually leave the epithelial layer, pass through the underlying basement membrane and accumulate in the interstitium of the tissue [61]. Here, these cells shed all of their epithelial markers and gain a full fibroblastic or mesenchymal phenotype. In a human study involving 133 patients with renal fibrosis, an EMT process was demonstrated in a significant number of samples as evaluated by a double-labeling microscopic technique for the markers cytokeratin, zona occludin 1 (ZO-1), vimentin and α-SMA [62]. 

TGF-β, MCP-1 and MMP-2 may identify patients at risk for renal fibrosis [63]. TGF-β may participate in renal fibrosis since TGF-β levels increase in fibrotic tissue from patients with unilateral ureteral obstruction. Smad3 is a signaling molecule downstream of the TGF-β receptor. In Smad3-deficient mice, renal fibrosis is abated, which indicates that inhibition of the TGF-β signaling pathway may provide a promising strategy to treat renal fibrosis [64]. In this regard, systemic injection of BMP7 (a TGF-β antagonist) reverses renal fibrosis in mice [65] and paricalcitol (a synthetic vitamin D analog that suppresses the expression of TGF-β and the Type I TGF-β receptor) attenuates ureteral obstruction-induced renal fibrosis in mice [66].

During fibrosis, endothelial cells associated with the microvasculature contribute to the formation of mesenchymal cells via a pathological EMT process called an endothelial-mesenchymal transition (EndMT) [67]. The EndMT resembles a normal, non-pathological EMT that occurs during embryonic development [68]. 

## 5. EMT and Cancer

During oncogenesis, the overexpression of various EMT signatures changes the epithelial cell polarity so that epithelial cells acquire morphological and biochemical traits indicative of mesenchymal cells. Under this pathological condition, the ability of the mesenchymal-like cancer cells to circumvent apoptosis, anoikis, cellular senescence and immune surveillance is linked to numerous EMT-associated genes. 

The EMT process facilitates the metastatic dissemination and progression of cancer cells from the site of the primary tumor to the neighboring tissues and distant organs. Although tumor heterogeneity and tumor plasticity have been long recognized, it is only in the last two decades that the EMT process has been identified as the important initial step and the underlying process for tumor metastasis [69,70], even though it was initially met with considerable skepticism. Although an EMT process was documented in many in vitro and in vivo cancer cell models, the role and relevance of an EMT process in human cancer remained a matter of debate. This debate was fueled due to inability to unambiguously identify an EMT process in human tumor samples since human tumors display a high degree of cell diversity and are difficult to follow in a temporal fashion. Today, the role of an EMT in cancer is attracting considerable attention from oncologists because accumulating evidence indicates that an EMT is of paramount importance in a plethora of cancer-related events [12,71]. In this regard, a Type 3 EMT can convert primary cancer epithelial cells into invasive, metastatic mesenchymal cancer cells with enhanced mobility [4]. The Type 3 EMT associated with oncogenesis may be viewed as an aberrant type of EMT that occurs in normal embryonic development. A Type 3 EMT is essential for metastatic cancer cells to avoid apoptosis, anoikis, oncogene addiction and cellular senescence. In addition, other EMT-driven cell machinery orchestrates immunosuppression in order to escape immune surveillance and remodels the ECM to complete the progression towards metastasis [27,72,73].

An EMT mediates tumor metastasis through a concert of cellular and molecular events rather than one single event. Many studies suggest that an EMT process occurring within cancer stem cells (CSCs) and circulating tumor cells (CTCs) characterizes a subpopulation of patients prone to relapse. In addition, CTCs with an EMT signature may co-exist or transform into relapse-initiating CSCs. Furthermore, an EMT process within cancer cells enables the remodeling of the ECM, which awakens the relapse-initiating CSCs [74].

### 5.1. EMT and Cancer Cell Survival

EMT-transformed cancer cells demonstrate a decreased proliferation (i.e., a quiescence phase) and an increased cell survival [75]. The entrance into a quiescent phase is determined by the activation of unfolded protein response pathways (e.g., eIF2α, p38α and TGF-β2) and the composition of the ECM [74,76]. In quiescent cancer cells, survival signals predominate over proliferative signals. The expression of Snail attenuates cell death (i.e., an anti-apoptotic response) in response to serum starvation and TNF-α treatment of Madin-Darby canine kidney (MDCK) cells [77]. This anti-apoptotic response is due to Snail expression associated with the activation of the MAPK/ERK and PI3K pathways. The association of an EMT process with enhanced cancer cell survival and resistance to apoptosis suggests a possible mechanistic explanation of why some tumors demonstrate a resistance to anti-neoplastic therapeutic strategies.

### 5.2. EMT and Disruption of Cell Junctions and Polarity

Cell surface protein complexes form epithelial cell-cell junctions that are essential for epithelial integrity. E-cadherin (an epithelial marker) is a transmembrane protein that adheres neighboring cells to one another and forms adherens junctions with its cytoplasmic tail linked to the actin cytoskeleton by α- and β-catenin. The loss of E-cadherin is a critical step in EMT process and a key feature of metastatic cells. Snail, Zeb and Twist (*E-cadherin* repressors) induce epigenetic silencing at the *E-cadherin* promoter region through hyper-methylation and histone de-acetylation [78]. Therefore, the loss of E-cadherin prevents the formation of adhering junctions that maintains the integrity of the epithelial layer and allows individual cells to roam freely, which is observed in cancer metastasis.

Vertebrate cells contact each other through tight junctions near the apical surface along with adherens junctions, desmosomes and gap junctions at the lateral surface [14]. These junctions weaken and their junctional proteins relocate or degrade upon initiation of an EMT process. The disruption of tight junctions during an EMT is accompanied by the decreased expression of claudin and occludin junctional proteins and the relocation of the zonula occludens 1 protein (ZO-1; also called TJP1). The de-stabilization of adherens junctions during an EMT is associated with the cleavage of E-cadherin at the cell membrane and its subsequent degradation [79]. As a result of this E-cadherin cleavage, β-catenin no longer interacts with E-cadherin and then is either degraded or protected from degradation in order to activate the downstream transcription [80]. The initiation of an EMT process also disrupts desmosomes and gap junctions. As an EMT progresses, the expression of junctional proteins is transcriptionally repressed, which secures the permanent loss of epithelial junctions [81].

The loss of epithelial cell polarity is another key step in the EMT process. Snail1 alters epithelial cell polarity by repressing Crumbs3 transcription and by abolishing Par and Crumbs3 localization at the cell junctions [82]. TGF-β contributes to the loss of cell polarity via the canonical pathway and non-canonical pathways. The canonical pathway induces *Snail* and *Zeb* gene expression. The non-canonical pathway down-regulates Par3 expression, degrades RhoA (Par6-mediated) and alters the actin cytoskeleton [83,84]. Par and Crumbs complexes, which are associated with Lin-7 localize apically in association with tight junctions and define the apical compartment; whereas, Scribble complexes define the basolateral compartment [85,86]. 

The loss of epithelial junctions during an EMT process results in a disruption of the apical-basal polarity. The decreased expression of E-cadherin in cancer cells prevents the interaction of SCRIB with the lateral cell membrane [87]. Furthermore, the decreased expression of either E-cadherin or SCRIB reduces cell adhesion and increases cell motility [88]. The change from apical–basal polarity to front–rear polarity involves Rho family small GTPases as well as apical–basal polarity proteins [89]. The decreased expression of CRB3 and LGL2 (polarity complex proteins) during an EMT process [90,91] further de-stabilizes the apical-basal polarity. 

### 5.3. EMT Confers Stem Cell Properties 

The EMT process has been closely linked to “stemness” in embryonic development and cancer [9]. Stem cells in normal tissues renew themselves through asymmetrical cell division while simultaneously generating committed progenitor cells that further differentiate to perform tissue-specific functions [92]. The study of neoplastic tissues has indicated the existence of self-renewing, stem-like cells within tumors that have been termed “cancer stem cells” (CSCs). Normal stem cells and CSCs share a common mesenchymal phenotype that enhances their ability to preserve stemness, to retain migratory properties and to respond to different stimuli during expansion and differentiation. CSCs were first identified in the hematopoietic system [93]. CSCs constitute a rare subset of cancer cells within a tumor and are defined by their ability to seed new tumors with a few cells. For this reason, CSCs have also been termed “tumor-initiating” cells [92]. CSCs have been linked to EMT phenotypes by epigenetic programming in many types of cancer. CSCs are found in solid tumors, including tumors in the breast, colon and brain [94,95]. Although CSCs are a small cell population in the tumor, CSCs may act as the driving force behind tumorigenesis and metastasis. CSCs resemble stem cells in normal tissues in that CSCs possess the ability to self-renew and to produce differentiated progeny cells with limited proliferative potential [96,97].

The EMT process enables cancer cells to disseminate and to self-renew during tumor metastasis. Untransformed immortalized human mammary epithelial cells undergo an EMT process upon Snail1 expression, Twist expression, or the presence of TGF-β1. The subpopulation of CD44 high/CD24 low immortalized human mammary epithelial cells that possess stem-like properties increases with concomitant induction of EMT phenotype. This finding indicates that an EMT process generates cells with many properties commonly observed in self-renewing stem cells. In this regard, it appears that the EMT process that enables cancer cell to disseminate from a primary tumor (i.e., metastasis) also promotes cancer cell self-renewal [98]. Zeb1 confers stem cell-like properties, which reinforces the connection between the EMT process and stemness [99]. In addition, numerous studies have demonstrated that a subset of isolated CSCs expresses EMT phenotypes in different cancers [100,101]. However, no appropriate marker is available to identify these EMT-transformed CSCs at present time. The identification of these EMT-transformed CSCs is important in order to avoid cancer relapse.

The transition of differentiated cancer cells into CSCs allows oncogenic mutations that arose in the differentiated cancer cells to integrate into the CSCs through the EMT process. The CSCs with the integrated oncogenic mutations undergo clonal expansion following cell invasion and dissemination promoted by the EMT process [102]. 

### 5.4. EMT and Circulating Tumor Cells

Circulating tumor cells (CTCs) play a major role in the design of therapeutic regimens and in the assessment of cancer progression. The presence of EMT CTCs indicates the risk of tumor relapse. The increased number of EMT CTCs correlates much more closely with prognosis of hepatocellular carcinoma patients than epithelial CTCs detected by EpCAM and cytokeratin [103]. In addition, the number of EMT CTCs detected by vimentin and twist increases during the progression of breast cancer through the early and late metastatic stages [104]. The novel marker Plastin3 was found to identify the most aggressive EMT CTCs in 33% of 711 colorectal cancer patients. These Plastin3-positive CTCs also showed staining for the EMT marker vimentin [105].

The number of CTCs has been used as an indicator of cancer progression and patient survival in more than 300 clinical trials [106]. The changes in the number of CTCs may indicate whether to maintain the current course of chemotherapy or to switch the course of chemotherapy. Since most of the above-mentioned clinical trials used EpCAM as a marker, which only identifies epithelial CTCs, an accurate assessment of the pharmacodynamics of the chemotherapeutic drug under trial may not have been achieved. In this regard, the use of Plastin3 and CSV (Cell-Surface Vimentin) as markers to identify EMT CTCs in addition to EpCAM is imperative to obtain a more accurate assessment of the role of CTCs in cancer progression and patient survival [74].

Recent studies have validated the existence of stem cell–like CTCs, which have the ability to self-renew, clonally expand and initiate tumors much like CSCs. These cells are EpCAM-positive CTCs and express CD133 and ABCG2 (CSC markers) and have the nuclear localization of β-catenin [107,108]. This suggests that EpCAM-positive CTCs with stem cell-like properties represent a subset of CTCs that possess a more aggressive phenotype leading to an earlier tumor relapse and a decreased patient survival. A possible strategy to isolate and enrich the CSC population may be to use CTC markers, which identify EMT-positive cell populations since we know that EMT-positive cancer cells predict aggressive tumor relapse in cancer patients [74]. Snail and Twist expression in mammary epithelial cells induces the formation of a mesenchymal cell population with a CD44 high/CD24 low phenotype, which is similar to an epithelial stem cell phenotype [98]. Differentiated cancer cells can transition into CSCs (and vice versa), which is consistent with the reversible nature of an EMT. This transition of differentiated cancer cells into CSCs enables oncogenic mutations present in the differentiated cancer cells to be passed down and integrated into the CSCs. The CSCs with new oncogenic mutations can now undergo clonal expansion after invasion, dissemination and the occurrence of an MET at a secondary tumor site [102,109]. 

The expression of TGF-β1 and TβRII in breast cancer CSCs is elevated compared to more differentiated breast cancer cells. In addition, the inhibition of the TGF-β signaling pathway in CSCs re-establishes the epithelial phenotype [110]. High levels of Wnt signaling and the nuclear localization of β-catenin have been described in colon CSCs [111]. Notch signaling participates in the generation of CSCs in many cancers, including pancreatic adenocarcinomas. In this regard, the inhibition of Notch signaling suppresses the EMT process and the formation of CSCs in a xenograft model [112,113].

CTCs with stem cell-like properties require further study in order to predict the tumor recurrence and determine the window of therapeutic treatment so that increased patient survival can be achieved. Using the next generation gene sequencing, CTC profiles may provide a powerful clinical tool to assess the change from a chemotherapeutic susceptible tumor to a chemotherapeutic resistant tumor. 

### 5.5. EMT and Drug Resistance to Cancer Therapy

EMT enhances tumor cell migration and invasion, which then contributes to poor patient survival. In addition, EMT induces drug resistance, metastasis, and stem cell-like properties in tumor cells. Drug resistant ending in tumor relapse and metastasis remains a major cause of morbidity and mortality in cancer patients despite successful surgical resection or chemotherapy. This is mainly due to the fact that tumor cells undergo dynamic clonal evolution in response to the selective pressure of therapeutic intervention. It has been hypothesized that the primary cause of tumor relapse and metastasis is due to the presence of CSCs, which not only express high levels of multidrug-resistant membrane transporters but also possess abnormally activated anti-apoptotic signaling cascades [114,115,116].

Tumor cells in the non-CSC population can de-differentiate to CSCs and spontaneously undergo EMT changes. These de-differentiated cells express CSC-like cell-surface markers and acquire an enhanced capacity to invade and metastasize in mice [97,117,118]. An EMT lineage-tracing system using a mesenchymal-specific Cre-mediated fluorescent marker switch system has been used to monitor the EMT process in spontaneous breast-to-lung cancer metastasis mouse models [117]. The EMT lineage-tracing system results indicated that only a small proportion of tumor cells within the predominant primary breast tumor undergo an EMT. Interestingly, lung metastasized breast cancer cells from the primary breast tumor do not undergo an EMT. However, cyclophosphamide-treated mice develop a significant number of EMT cancer cells during the lung metastasis [117]. More importantly, EMT cells significantly contribute to recurrent lung metastasis formation after chemotherapy. The above-mentioned findings demonstrate that an EMT plays an important role in cancer drug resistance and contributes to metastasis after chemotherapy treatment. 

The EMT-driven drug resistance has been attributed to various mechanisms. One of the main mechanisms is excessive drug efflux by cell membrane transporter proteins, especially the ATP-binding cassette (ABC) transporter family that expresses an efflux pump [114]. Normal tissues derive protection from drug toxicity due to the efflux action of ABC transporters [119]. However, cancer cells develop drug resistance due to the efflux action of ABC transporters. Selection of cancer cells in vitro with anticancer drugs (e.g., paclitaxel, doxorubicin, or vincristine) frequently results in multidrug resistance due to increased ABC transporter expression [114]. Vincristine-resistant human colon adenocarcinoma cells acquire a mesenchymal phenotype along with an enhanced migratory and invasive ability both in vitro and in vivo. In this regard, Twist1 (a major EMT inducer) significantly increases in vincristine-resistant human colon adenocarcinoma cells. Interestingly, Twist1-silencing reverses the EMT phenotype and enhances the chemo-sensitivity to anticancer agents including vincristine suggesting that EMT promotes the vincristine resistance of colon adenocarcinoma cells through Twist1 [120]. Moreover, Twist, Snail and Foxc2 (EMT-inducing transcription factors) increase ABC transporters, which leads to multidrug resistance in invasive breast cancer cells [115]. 

Another important mechanism underlying EMT-driven drug resistance involves resistance to apoptosis. Breast cancer cells that survive cyclophosphamide treatment undergo an EMT, which accompanies an apoptotic tolerance and increased expression of chemoresistance-related genes [117]. Urothelial carcinoma cells that acquire cisplatin resistance demonstrate a low apoptotic frequency and pronounced G2/M arrest [121]. CSCs express high levels of anti-apoptotic proteins that protect CSCs from apoptosis [122]. B-cell lymphoma 2 (Bcl-2) is the major inhibitor of apoptosis in any animal species [123]. Bcl-2 proteins decrease mitochondrial apoptosis by stabilizing mitochondrial permeability. The pro-apoptotic proteins (Bax, Bak and Bcl-xS) or anti-apoptotic proteins (Bcl-2, Bcl-xL and Mcl-1) regulate Caspase-9 and -3 activities leading to apoptosis. Repeated cisplatin treatment of epithelial ovarian tumors leads to formation of CSCs and suppression of Bcl-2/Bcl-xL [124]. Human non-small cell lung cancer cells that survive cisplatin treatment undergo an EMT. Such EMT-associated chemoresistance is mediated through the Mcl-1-dependent anti-apoptotic pathway [125]. Furthermore, normal epithelial cells undergo anoikis (a form of apoptosis) due to the loss of anchorage to the substratum. In this regard, integrins play an essential role in the attachment of epithelial and endothelial cells to the extracellular matrix (ECM). Hence, integrin-dependent adhesion to ECM components promotes cell survival [116]. Integrin-mediated epithelial cell-ECM interactions are mediated by active focal adhesion kinase (FAK) and its downstream signal Src, which confers anoikis resistance to pancreatic carcinoma, prostate cancer, along with metastatic breast and lung cancer cells [116,126]. 

In recent years, the role of EMT in the development of drug resistance has gained increasing attention with efforts to target EMT in order to increase the sensitivity of cancer treatment. Salinomycin (an antibacterial and anticoccidial drug) improves doxorubicin sensitivity by reducing drug efflux pump expression and activity in breast cancer cells [127]. Carnosol (a natural inhibitor of MDM2/p53 complex) controls TGF-β-induced EMT master regulator genes (*Slug*, *Snail*, *Twist* and *Zeb1*) and increases the treatment sensitivity of temozolomide in human glioblastoma [128]. Resveratrol (a red grape skin extract) exhibits an anti-proliferative property by inducing mitochondrial apoptosis and by inhibiting EMT in oral squamous carcinoma cells [129]. Shikonin (an active compound from *Lithospermum erythrorhizon*) reverses hepatocyte growth factor (HGF)-induced migration, invasion and an EMT marker change (e.g., a decrease in vimentin, Slug and Snail expression) [130]. 

In conclusion, EMT significantly contributes to the development of drug resistance in multiple cancer types. The mechanism of EMT-driven drug resistance remains largely obscure. Moreover, many current studies that show improvement in drug resistance by targeting EMT are based on in vitro cancer cell lines or in vivo animal models. Consequently, more studies that focus on clinical patient application are needed. Despite limitations, the potential of an anti-EMT approach as an adjunct to current antitumor therapies remains optimistic. In this regard, EMT suppression together with chemotherapy or targeted therapy drugs may potentially serve to improve the sensitivity of current cancer therapeutics. 

## 6. Key Regulatory Molecular Mechanisms of EMT 

There are a number of distinct molecular mechanisms that initiate an EMT process and bring the EMT process to completion. Cells involved in an EMT process down-regulate the expression of epithelial proteins (e.g., cell junction proteins) and redirect their gene expression profiles in order to promote changes in the cytoskeleton and to alter their interaction with the ECM. In many cases, the factors produced by this redirection of gene expression profiles provide valuable biomarkers to identify various stages as the cell passages through an EMT. 

### 6.1. Gene Expression Changes in EMT

The loss of *E-cadherin* gene expression is an important event in an EMT and also in the destabilization of adherens junctions. Consequently, many studies have focused on the mechanism by which *E-cadherin* is regulated during cancer progression. Factors that induce the EMT process are classified based on their ability to repress *E-cadherin* directly or indirectly (Table 2).

In addition, the repression of *claudin* and *occludin* gene expression promotes the disruption of apical tight junctions. Whereas, the repression of *desmoplakin* and *plakophilin* gene expression promotes the disruption of desmosomes [14]. This repression of gene expression prevents the de novo formation of cell-cell junctions and results in the disruption of the epithelial cell layer [131]. The above-mentioned repression of gene expression for cell junction proteins is accompanied by the activation of gene expression for proteins expressed in mesenchymal cells. 

N-cadherin connects to the cytoskeleton through α-catenin and β-catenin. In addition, N-cadherin interacts with p120, various signaling molecules and receptor tyrosine kinases (RTKs) such as platelet-derived growth factor (PDGF) and fibroblast growth factor receptors (FGFRs) [79,132,133]. N-cadherin also interacts with the neural cell adhesion molecule (NCAM) during the EMT process [79,134]. NCAM modulates the activity of various RTKs and FYN (a SRC family tyrosine kinase), which facilitates the assembly of focal adhesions, cell migration and cell invasion [135]. 

Changes in gene expression for cytoskeletal proteins and cell polarity complex proteins also occur during the EMT process. In the first example, the composition of intermediate filaments changes during the EMT process as evidenced by the repression of the *cytokeratin* gene and the expression of the *vimentin* gene [14]. Keratin and vimentin intermediate filaments both regulate trafficking of organelles and membrane-associated proteins; but keratin and vimentin traffic different membrane-associated proteins to the cell membrane [136]. In the second example, the direction of cell motility changes during the EMT process as evidenced by the repression of the *Crumbs3* gene, *PATJ* gene and the *LGL2* gene. Crumbs3 and PATJ are apical Crumbs complex proteins and LGL2 is a basolateral Scribble complex protein [79,89,137]. 

As epithelial cells differentiate into mesenchymal cells, the epithelial cells lose their interaction with the basement membrane and begin to communicate with the underlying ECM. This means that the epithelial cells down-regulate some epithelial integrins but up-regulate other integrins that play a key role in the EMT process [79]. The changes in the integrin profile during an EMT correlate with the increased expression of proteases (e.g., MMP2 and MMP9 matrix metalloproteinases) that enhance protein degradation and promote cell invasion. MMPs also target transmembrane proteins (e.g., E-cadherin), which results in shedding of the extracellular domain of E-cadherin and the disruption of adherens junctions [138].

### 6.2. Complexity in EMT Signaling Pathways

Various extracellular ligands activate and maintain the EMT process in either an autocrine or paracrine manner. EMT cell culture models facilitated the characterization of complex molecular mechanisms associated with an EMT and have revealed crosstalk between EMT signaling pathways and transcriptional, translational and post-translational regulation [9]. There are at least four signaling pathways that operate in early embryonic development, which include: (1) Receptor tyrosine kinase (RTK) pathway; (2) TGF-β family pathway; (3) Wnt pathway; and (4) Hedgehog family pathway. These signaling pathways are much conserved and the protein components of these pathways date back 2–3 billion years [147]. Thus, it is of no surprise that some of these signaling pathways play an important role in early embryonic development and in the EMT process. Moreover, these signaling pathways are usually response to various inductive stimuli and activate the EMT inducing transcriptional factors, such as Snail1/2, Twist, Zeb1/2, which serve as major signaling mediators that initiate and maintain the process of EMT.

RAS/MAPK/ERK and NFκB are the major downstream signals of receptor tyrosine kinase. Hepatocyte growth factor (HGF)/Scatter factor (SF) can activate the RAS/MAPK/ERK signaling pathway with the cooperation of EGF-1 (early growth response factor-1) that binds to the *Snail1* promoter, leading to the induction of Snail1 [148]. 

Snail1 can be phosphorylated by multiple kinases. Glycogen synthase kinase-3β (GSK3β) is involved in Snail1 phosphorylation at two serine-rich motifs. This phosphorylation facilitates the nuclear export of Snail1 as well as enables ubiquitin-mediated degradation of Snail1, which inactivate Snail1 [149]. Phosphorylation of Snail1 by protein kinase D1 (PKD1) also facilitates nuclear export of Snail1 [150]. Conversely, phosphorylation of Snail1 by p21-activated kinase 1 (PAK1) or large tumor suppressor (LATS2) facilitates the nuclear retention of Snail1 and enhances the activity of Snail1 and promotes EMT [151,152]. Likewise, the de-phosphorylation of Snail1 by small C-terminal domain phosphatase 1 (SCP1) also facilitates the nuclear retention of Snail1 and enhances the activity of Snail1 [153]. Snail2 is degraded when Snail2 undergoes a p53-mediated recruitment to the mouse double minute 2 (MDM2) complex [154]. 

Snail1 modulates the EMT process and represses its own transcription [155] while Snail2 self-activates in avian neural crest cells [156]. Snail1 also cooperates with ETS1 (activated by MAPK/ERK) to upregulate MMP expression [157]. MAPK/p38, JUN N terminal kinase (JNK) and MAPK/ERK phosphorylates Twist and protect Twist from degradation, facilitates nuclear import of Twist, and thus promotes EMT [158]. The NFκB signaling pathway induces Snail1 transcription and protein stabilization, which regulates the EMT process in carcinoma cell lines and mesothelial fibrosis [159,160,161]. The involvement of the NFκB pathway in the EMT process is substantiated since non-destructible IkB (an NFκB inhibitor) blocks the EMT process [162]. 

The TGF-β signaling pathway operates with TGF-βR1 and TGF-βR2, which phosphorylate Smad2 and Smad3. The phosphorylated Smad2 and Smad3 form complexes with Smad4 to regulate the expression of genes that induce EMT [163,164]. Smad2 and Smad3 are also involved in histone modifications by associating with epigenetic regulators, such as Trim33, to create an easy access to transcriptional regulators [165]. In addition, TGF-β reduces the level of the heterochromatin mark H3 Lys9 dimethylation and increases the levels of euchromatin mark H3 Lys4 trimethylation and the transcriptional mark H3 Lys36 trimethylation in mouse hepatocytes [166]. The changes of these histone modification marks are necessary for the appearance of EMT-mediated phenotypes. NFκB, which upregulates Zeb1/2, binds regulatory gene sequences at E-boxes and repress or activate transcription [131,167]. Zeb-mediated transcriptional repression often involves the recruitment of a C-terminal-binding protein (CTBP) co-repressor. However, Zeb1 represses *E-cadherin* expression independently of CTBP by recruiting the Switch/sucrose non-fermentable (SWI/SNF) chromatin remodeling protein BRG1 [168,169]. The induction of *Zeb1* expression involves a cooperation between Twist1 and Snail1 [170].

The importance of Wnt pathway has been discussed in the gastrulation and neural crest formation. In tumor cells, in the absence of Wnt signals, GSK3β stays free and active, which can phosphorylate its downstream signal β-catenin for protein degradation. In the present of Wnt signals, GSK3β is phosphorylated and inactivated. Thus, β-catenin is free in the cytosol and further transported into the nucleus. In the nucleus, with the help of transcriptional promotor, TCF/LEF, β-catenin is a transcriptional factor and activates the transcription of Snail1 [171]. Snail1 can form a positive feedback by direct binding with β-catenin [172].

The Sonic hedgehog (SHH) pathway has “Off” and “On” states. In the absence of SHH, the pathway is at an “off” state. Glioma-associated oncogene homologs (Glis), a key molecule in SHH pathway, has three isoforms. Gli1 is transcriptionally repressed, Gli2 is phosphorylated by GSK3β and CK1 for degradation, and Gli3 is processed to a cleaved repressor [173]. In the present of SHH, the pathway is at an “on” state. Glis are stabilized and enter cell nuclei [173]. Activated Gli1 upregulates Snails and Twist, hence promotes EMT [174,175].

Understanding the role of specific signaling pathway facilitates the identification of therapeutic targets for EMT. Thanks for the significant progress that scientists made in the past decades, the function of many signaling pathways has been uncovered. However, signaling pathways do crosstalk with each other and function in both independent and dependent mechanisms. For example, Hedgehog signals induces JAG2 for Notch mediated Snail1 upregulation and also induces TGFß1 secretion for Zeb1 and Zeb2 upregulation [171,173]. In addition, SHH pathway suppresses Wnt pathway by stimulating Wnt antagonist, SFRP1, and these two pathways share the common enzyme, GSK3β [176]. These studies indicating that the complex interaction among cell signaling pathways need to be seriously considered during drug development. Due to the complexity of signaling pathway networks, further effort is needed to elucidate their relationships.

### 6.3. EMT and MicroRNAs 

MicroRNAs (miRNAs) are small 18–24 nucleotide non-coding RNAs that induce mRNA degradation or translational repression through specific base pairing, typically within the 3′ UTR [177]. The miRNAs have been implicated in many cell processes, including the regulation of cancer progression and the EMT process. For example, the up-regulation of miR-9 directly targets E-cadherin, which leads to increased cell motility and cell invasion in breast cancer cells [178]. In addition, the overexpression of miR-9 causes non-metastatic breast cancer cells to form pulmonary micro-metastases in mice. Conversely, the inhibition of miR-9 by using a “miRNA sponge” prevented metastasis in highly malignant cells. The expression of miR-495 promotes colony formation in vitro and tumorigenesis in vivo in breast cancer cells [179]. Both miR-495 and miR-9 promote cell invasion and oncogenesis via the direct suppression of E-cadherin. Many studies have demonstrated that the miRNAs induce gene expression through promoter binding, which adds to the complexity of miRNA action. In this regard, it has been reported that miR-373 binds and activates the *E-cadherin* promoter [180]. 

MiRNAs also regulate hallmark proteins of an EMT process (e.g., vimentin, N-cadherin). For example, miR-30a directly targets vimentin and thereby inhibits cell migration and cell invasion in breast cancer. Furthermore, the underexpression of miR-30a in breast cancer cells from patients was associated with unfavorable outcomes (e.g., a later tumor stage, lymph node metastasis) and even worse outcomes (e.g., increase recurrence rates, decreased long-term survival). This suggests that miR-30a may serve as breast cancer prognostic marker [181]. MiR-138 and miR-17-3p target vimentin and thereby demonstrate tumor-suppressive or anti-neoplastic effects [182]. This is highlighted by the findings that miR-17-3p inhibits the expression of vimentin in prostate cancer and that miR-17-3p expression in prostate cancer specimens and cell lines inversely correlates with aggressiveness. In other words, a low expression of miR-17-3p correlates with a high tumorigenesis and metastasis while a high expression of miR-17-3p correlates with a low tumorigenesis and metastasis. The miR-17-3p expression also inversely correlates with an increased prostate cancer Gleason score. Finally, miR-17-3p restoration blocks prostate tumor growth in male athymic nude mice, which indicates that miR-17-3p may function as a tumor suppressor in prostate cancer [183]. 

MiRNAs also regulate N-cadherin through 3′ UTR regulation. MiR-145 directly targets N-cadherin and thereby suppresses gastric cancer cell migration and invasion in vitro. MiR-145 inhibits metastasis in vivo, which confirms the role of miR-145 in suppressing the invasion-metastasis cascade [184]. Besides N-cadherin, miR-145 targets 3′-UTRs of several other genes involved in an EMT and cancer metastasis. MiRNAs target various transcriptional factors involved in the EMT process; that is, miR-30 targets Snail, miR-124 targets Slug, and miR-200 targets Zeb1 and Zeb2 [185,186]. Numerous studies have demonstrated that miRNAs impact tumor plasticity and the EMT process at various cell levels and through various mediators. This highlights the significance and importance of miRNAs and elucidating their role in complex interactions. 

### 6.4. EMT and Autophagy

Although EMT and autophagy are two different biological processes in cancer, recent studies indicate that these two processes can interact with each other and be influenced by common signaling pathways. Autophagy occurs when the cells are under environmental stress, such as hypoxia, nutrient deprivation and drug treatment [187]. Autophagy allows the degradation and recycling of cellular components in an unfavorable condition and then uses these cellular components as “building blocks” for other cells. Thus, autophagy provides a strategy for cancer cells to survive under the harsh conditions. During the EMT, cancer cells undergo the metabolic reprogramming to acquire the metastatic status and support the energy requirements [188]. Both autophagy and EMT are regulated by p53 and RAS signaling pathways during cancer progression [189]. p53 and RAS regulate autophagy through the action of autophagy related genes (ATG) to form autophagosomes [190]. TGFβ is another important signaling pathway that can activate both EMT and autophagy processes. TGFβ promotes autophagy and cell death during the early stage of tumor progression. However, TGFβ induces EMT and promotes cancer cell metastasis during the late phase of tumor progression, [187]. 

There are studies indicating that autophagy may assist EMT cancer cells during the metastasis since autophagy supports the cell viability [187]. Autophagy also assists EMT cancer cells to escape from immune surveillance in breast carcinoma [191]. However, other studies showed that induction of autophagy reverses EMT and inhibits cancer cell migration and invasion [192]. These dual effects of autophagy on EMT demonstrate the complexity of their interactions, which may depend on the type and the stage of the cancer. 

## 7. Concluding Remarks 

Genes that play a critical role in embryonic development are frequently culprits in oncogenesis. Conversely, genes that play a critical role in oncogenesis are frequently key players in embryonic development. This relationship between embryonic development and oncogenesis applies to steps that initiate tumor formation, tumor progression and metastasis. As one would expect, genes implicated in the EMT process during embryonic development have also been implicated in oncogenesis. 

Current chemotherapeutic treatments predominately target the cell proliferation capability of the cancer cell. Although it may seem reasonable to treat a proliferative disease like cancer with anti-proliferative drugs, one must remember that the cell proliferation capability of all cells is staunchly protected. In this regard, billions of years of evolution has resulted in cell proliferation becoming the most protected, most redundant and most firmly entrenched capability compared to all other cell capabilities such as cell differentiation and embryonic morphogenetic movement. The robust and redundant protections for cell proliferation are evolutionarily much older than the more recent capabilities of cell differentiation and its regulation that evolved with multicellular eukaryotes. Accumulating data on CSC and drug resistance studies, as discussed above, indicate that EMT and MET may be the real Achilles heel of cancer. Consequently, it would also seem reasonable to target EMT and MET as additional therapeutic options to treat cancer.

## Figures and Tables

**Table 1:** Three types of the epithelial mesenchymal transition.

Types of EMT	Functions	Features	Consequences
I	Implantation, embryogenesis and organ development	Generates diverse cell types that may undergo an MET to form secondary epithelial cells during embryogenesis	No fibrosis; No invasion
II	Wound healing, tissue regeneration and organ fibrosis	Generates fibroblasts and other related cells to reconstruct tissues following trauma and inflammatory injury	Fibrosis; No invasion
III	Malignant transformation of cancer cells	Produces cancer cells that maintain many epithelial traits along with some mesenchymal traits or produce cancer cells that become fully mesenchymal	Invasion and metastasis

**Table 2:** List of EMT inducers that repress E-cadherin.

	Molecules	Description	References
Direct E-cadherin repressors	Snail1 (Snail), Snail2 (Slug), Snail3 (Smuc)	Zinc finger proteins of the *Snail* superfamily	[139]
	Zeb1 (δEF1), Zeb2 (SIP1), Tcf8	Zinc finger and E-box binding proteins of the *Zeb* family	[140]
	E47	A βHLH factor	[141]
	Klf8	A Krüppel-like factor	[142]
Indirect E-cadherin repressors	Twist1, Twist2	Twist βHLH proteins	[131]
	GSC and Six1	Homeobox proteins	[143,144]
	E2.2	A βHLH factor	[145]
	Foxc2	A forkhead-box protein	[146]
